# Redefining Pituitary Neuroendocrine Tumors in MEN1: Prevalence, Clinical Behavior, and Implications for Long-Term Surveillance

**DOI:** 10.3390/curroncol33020097

**Published:** 2026-02-04

**Authors:** Roberta Modica, Alessia Liccardi, Roberto Minotta, Elio Benevento, Gianfranco Di Iasi, Massimo Di Nola, Michele Coletta, Annamaria Colao

**Affiliations:** Endocrinology, Diabetology and Andrology Unit, Department of Clinical Medicine and Surgery, Federico II University of Naples, 80131 Naples, Italy; roberto.minotta@unina.it (R.M.); elio.benevento@unina.it (E.B.); g.diiasi@studenti.unina.it (G.D.I.); massimo.dinola@unina.it (M.D.N.); michele.coletta@unina.it (M.C.); colao@unina.it (A.C.)

**Keywords:** MEN-1, multiple endocrine neoplasia type 1, neuroendocrine tumors, pituitary adenoma

## Abstract

Background: Pituitary neuroendocrine tumors (PitNETs) are a core manifestation of multiple endocrine neoplasia type 1 (MEN1), yet their true prevalence, biological behavior, and optimal management remain debated. Earlier reports suggested increased aggressiveness compared with sporadic PitNETs, while more recent surveillance-based studies indicate a predominantly indolent phenotype. Methods: We conducted a retrospective single-center study including all patients with clinical, familial, or genetic MEN1 referred to the Endocrinology Unit of the University of Naples “Federico II”, ENETS Center of Excellence, between January 2004 and June 2025. Demographic, clinical, radiological, hormonal, and therapeutic data were systematically collected. PitNETs were classified by size and hormonal activity. Results: Among 103 MEN1 patients (61 women), 50 (48.5%) were diagnosed with PitNETs at a mean age of 35.1 years. Microadenomas predominated (60%), and tumors were equally distributed between functioning and non-functioning lesions. Prolactin-secreting PitNETs were the most common functioning subtype (42%), followed by rare GH-, ACTH-, or mixed-secreting PitNETs. Dopamine agonists, mainly cabergoline, were prescribed in 38% of cases, while neurosurgical intervention was required in 14%, exclusively for macroadenomas. During follow-up, recurrence occurred in 8% of patients. No significant sex-related differences were observed in prevalence, tumor size, functional status, treatment approach, or outcomes. Conclusions: In our MEN1 cohort, PitNETs were frequent but largely indolent, with a predominance of microadenomas and limited need for surgery. Our findings support individualized, subtype-driven surveillance strategies, with conservative management for clinically non-functioning microadenomas and closer monitoring of prolactin-secreting PitNETs due to variable medical responsiveness.

## Simple Summary

Multiple endocrine neoplasia type 1 is a rare inherited condition that causes tumors in several hormone-producing glands, including the pituitary gland. In this study, we analyzed pituitary tumors in over 100 patients followed at a single specialized center over two decades. Almost half of the patients developed a pituitary tumor, most often small lesions detected through regular screening. About half of the tumors produced excess hormones, mainly prolactin, while the others were hormonally inactive. Most tumors were successfully managed with medication or careful monitoring, and surgery was required only in a minority of cases, usually for larger tumors. Tumor recurrence was uncommon, and no meaningful differences were observed between men and women. Overall, our findings suggest that pituitary tumors in this condition are generally less aggressive than previously thought. These results support personalized follow-up strategies, with less intensive monitoring for inactive small tumors and closer surveillance for hormone-secreting ones.

## 1. Introduction

Multiple endocrine neoplasia type 1 (MEN1) is a rare autosomal dominant disorder characterized by the development of multiple endocrine and non-endocrine tumors, most notably involving the parathyroid glands, pancreatic islets, and pituitary gland [1,2]. This rare syndrome has an incidence of 0.22–0.25% and a prevalence of 1–10/100,000 live births. Pituitary neuroendocrine tumors (PitNETs) represent one of the three hallmark manifestations of MEN1 and are considered a classic diagnostic criterion of the disease [1,2,3]. Their reported prevalence ranges from 10% to 60%, with up to 65% in autopsy series, suggesting that pituitary involvement is often underdiagnosed [1]. Although the median age at diagnosis is around 40 years, PitNETs may present much earlier, with cases reported in children as young as 5 years of age [1]. Apparently, there are no sex-related differences in terms of incidence and aggressiveness of PitNETs in the context of MEN1 [4].

According to the most recent World Health Organization (WHO) classification, tumors previously referred to as “pituitary adenomas” are now designated as PitNETs, reflecting their neuroendocrine origin and a classification framework more closely aligned with other neuroendocrine neoplasms [5]. The current WHO classification emphasizes tumor lineage determination based on immunohistochemical expression of pituitary-specific transcription factors—namely PIT1, TPIT, and SF1—followed by hormone immunostaining, allowing for a more precise biological and prognostic characterization [5]. This updated approach has also refined the definition of clinically nonfunctioning tumors and has restricted the diagnosis of true null cell PitNETs to lesions lacking both hormonal expression and lineage-defining transcription factors [5].

Historically, MEN1-related PitNETs—particularly prolactinomas—were considered to display a similar clinical behavior and therapeutic response to sporadic lesions [3]. However, evidence from large multicenter cohorts, including the French and Belgian MEN1 series and the French Group of Endocrine Tumors (GTE), indicated that PitNETs in MEN1 patients tend to be larger, more invasive, and less responsive to conventional treatments [6,7,8]. These findings initially supported the concept of an intrinsically more aggressive biological profile of MEN1-associated PitNETs. More recent studies, however, have challenged this view. Data from the Dutch MEN1 cohort, the Mayo Clinic, and updated GTE analyses have shown that the majority of PitNETs diagnosed through systematic MEN1 surveillance are small, clinically nonfunctioning microadenomas, with a clinical course resembling that of sporadic lesions [9]. This heterogeneity underscores the complexity of PitNET behavior in MEN1 and suggests that aggressiveness may have been previously overestimated due to diagnostic bias and referral patterns.

From a prognostic perspective, MEN1-associated PitNETs exert a multifaceted impact. Their clinical relevance derives from three major domains: tumor mass effect (with risk of visual impairment), hormonal hypersecretion (most commonly prolactin, but also growth hormone or ACTH), and treatment-related morbidity, particularly surgical complications [1]. Importantly, given the potential for earlier onset and underdiagnosis, international clinical practice guidelines recommend systematic biochemical screening and pituitary MRI from early childhood in MEN1 carriers, with periodic follow-up to enable timely detection and management [10,11]. Nonetheless, optimal surveillance strategies remain debated, especially in light of the increasing detection of incidental clinically nonfunctioning microadenomas and concerns regarding repeated gadolinium exposure during MRI monitoring [9,11,12].

In this context, MEN1-related PitNETs represent a distinct clinical and biological entity compared to sporadic PitNETs, with differences in age of onset, natural history, and treatment outcomes. Clarifying their true epidemiology, aggressiveness, and prognostic determinants remains critical, both for refining patient counseling and for guiding surveillance and therapeutic strategies in this complex hereditary syndrome. Furthermore, several studies addressing this topic have been conducted using large national registries. While such databases allow for the inclusion of greater sample sizes, they are inherently subject to limitations such as incomplete or inaccurate data reporting. For this reason, analyses from single institutions or multicenter collaborations remain essential, as they provide high-quality, real-life insights into the clinical management of this rare and heterogeneous group of neuroendocrine tumors.

## 2. Materials and Methods

All patients with a clinical, familial, or genetic diagnosis of MEN1 syndrome who were referred to the Endocrinology Unit of the University of Naples “Federico II,” an ENETS Center of Excellence for the diagnosis and treatment of neuroendocrine tumors (NETs), between January 2004 and June 2025, were included in this study. The diagnosis of MEN1 was established in patients who presented with at least two of the three major MEN1-associated lesions—primary hyperparathyroidism, enteropancreatic neuroendocrine tumors, or pituitary adenomas/PitNETs—regardless of the presence of a germline mutation. In addition, individuals carrying MEN1 germline mutations identified through familial screening were also considered to have MEN1, irrespective of whether they had developed clinical lesions at the time of evaluation. Individuals diagnosed with MEN4 syndrome were excluded.

Data were retrospectively collected on demographic and anthropometric variables, including body mass index (BMI), age at MEN1 diagnosis, and presenting clinical manifestations. BMI was calculated as weight in kilograms divided by height in meters squared (kg/m^2^). For patients with MEN1-related manifestations, additional information was recorded regarding age at onset of each manifestation, hormonal activity of neuroendocrine tumors, tumor size, and type of treatment, including the titration dose of medical therapy.

PitNETs were classified according to the histopathological and immunohistochemical criteria available at the time of diagnosis. In particular, hormone immunostaining was performed when surgical specimens were available. However, a systematic immunohistochemical panel for pituitary transcription factors (PIT1, TPIT, and SF1), as recommended by the most recent WHO 2022 classification of PitNETs, was not routinely applied throughout the entire study period, given the long retrospective timeframe of the cohort.

Accordingly, PitNETs were categorized as clinically functioning or clinically nonfunctioning based on clinical presentation, biochemical findings, and available hormone immunohistochemistry. Tumors classified as “clinically nonfunctioning” did not present with biochemical or clinical evidence of hormone hypersecretion. Due to the lack of systematic transcription factor analysis, a definitive diagnosis of null cell PitNET—defined by negativity for PIT1, TPIT, SF1, and pituitary hormones—could not be consistently established.

During follow-up, patients underwent standardized surveillance in accordance with current guidelines. This included routine biochemical assessments, measurements of plasma hormone levels, abdominal and pituitary magnetic resonance imaging (MRI), endoscopic ultrasonography of the abdomen, thoracic computed tomography (CT), and functional imaging with somatostatin receptor scintigraphy or ^68Ga-DOTATOC PET/CT. In line with international guidelines, dopamine agonist (DA) resistance was defined by the inability to achieve PRL normalization and a tumor shrinkage of at least 50% despite up-titration to doses ≥2.0 mg/week for cabergoline [13].

Descriptive statistics were generated for the study population. Baseline characteristics were compared between men and women using Fisher’s exact test for categorical variables and the Mann–Whitney U test for continuous variables. All analyses were performed using SPSS Statistics software, version 23.0 (IBM Corporation, Armonk, NY, USA).

The study protocol was approved by the Ethics Committee of the University of Naples “Federico II” (approval no. 259/23), and written informed consent was obtained from all participants.

## 3. Results

Among the 103 MEN1 patients included in the study (61 women and 42 men), PitNETs were identified in 50 individuals (48.5%). The mean age at diagnosis was 35.1 years, ranging from 10 to 72 years. Microadenomas were more common, occurring in 30 patients (60.0%), while 20 patients (40.0%) had macroadenomas. Clinically functioning tumors were detected in half of the cases (25/50), the majority being prolactin-secreting PitNETs (21 patients, 42.0%), with a nearly equal sex distribution. Less frequent were mixed GH/PRL-secreting PitNETs (2 patients, 4.0%), a single GH-secreting PitNET (2.0%), and a single ACTH-secreting PitNET (2.0%). The remaining 25 PitNETs (50.0%) were classified as clinically nonfunctioning. The mean lesion size was 9.4 mm (range 2–60 mm). Medical therapy with dopamine agonists was prescribed in 20 patients (38.0%), most of whom received cabergoline (*n* = 19), with a mean weekly titrated dose of 1.32 mg (range 0.5–4.5). Only one patient refused medical therapy. All patients undergoing DA medical therapy showed a reduction in PRL levels of more than 50% after 3 months. Endoscopic neurosurgery was performed in 7 patients (14.0%), all of whom presented with macroadenomas, and 6 were clinically functioning. Surgical indication was based on both dimensional and functional criteria; in case of acromegaly and Cushing-associated syndrome, surgery was the first-line therapy. Specifically, all surgically treated PitNETs showed symptoms related to a mass effect, predominantly visual disturbances, associated with tumor size and local compression. The majority of surgically treated patients were women (6 out of 7). According to the updated WHO classification of PitNETs and based on the available histopathological data, 4 surgically treated tumors were classified as probably null cell PitNETs, given the absence of immunoreactivity for all pituitary hormones. However, immunohistochemical evaluation of pituitary transcription factors (PIT1, TPIT, SF1, and GATA3) was not available, precluding a definitive lineage assignment. One patient was diagnosed with a corticotroph PitNET, while two patients were classified as mammosomatotroph PitNETs. During follow-up, disease recurrence was documented in 4 patients (8.0%), predominantly in women (*n* = 3). Radiological recurrence occurred after surgical treatment in 4 patients, among these 2 patients experienced biochemical recurrence too. Of these, 1 was managed with medical therapy, 3 required repeat neurosurgical intervention, and 1 also underwent adjuvant stereotactic radiotherapy (Table 1). When stratifying the cohort by sex, no significant differences emerged across the analyzed variables. Specifically, the incidence of PitNETs did not differ between males and females (*p* = 0.84), nor did the distribution of microadenomas versus macroadenomas (*p* = 0.62) or functioning versus non-functioning tumors (*p* = 0.48). Similarly, treatment resistance (*p* = 0.16), the proportion of patients undergoing surgical therapy (*p* = 0.23), age at diagnosis (*p* = 0.87), tumor size (*p* = 0.56), and the titration dose of cabergoline used (*p* = 0.11) showed no sex-related variation.

## 4. Discussion

**Figure 1:**
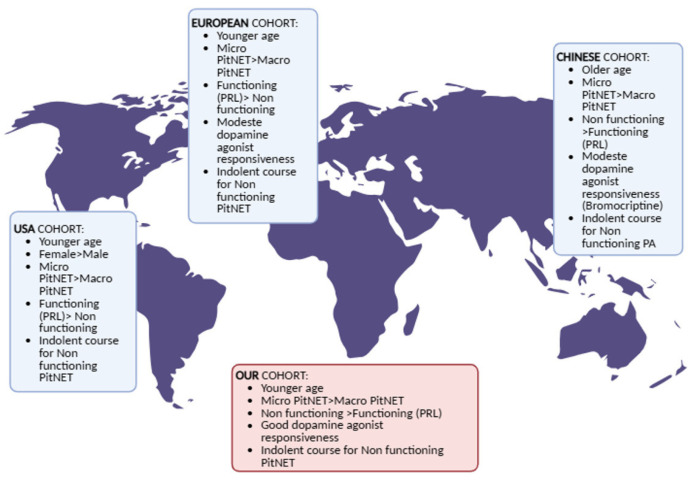
Worldwide Geographic Patterns of PitNET Clinical Manifestations.

This single-center retrospective study, conducted at an ENETS Center of Excellence, provides an updated analysis of MEN1-related PitNETs in a well-characterized Italian cohort, expanding upon the findings previously reported by the same Italian group [4]. The updated cohort includes 103 MEN1 patients, confirming a PitNET prevalence of 48.5%, consistent with that reported in previous multicenter studies and confirming the high frequency of pituitary involvement in MEN1. The mean age at diagnosis (35.1 years) also falls within the expected range, further supporting the representativeness of our cohort. Our findings indicate that microadenomas were more common than macroadenomas, occurring in 60% of patients, while functioning and non-functioning tumors were equally distributed. Among functioning tumors, prolactin-secreting PitNETs represented the vast majority, in line with prior reports that identify them as the most prevalent subtype in MEN1-related PitNETs. Less common entities, such as GH/PRL-secreting PitNETs, isolated GH-secreting PitNETs, and ACTH-secreting PitNETs, were observed only sporadically, confirming their rarity in this setting. Medical therapy with dopamine agonists was frequently employed, most commonly with cabergoline, with overall good tolerability and response. Neurosurgical intervention was reserved for macroadenomas, particularly in symptomatic cases, which is consistent with standard practice. Interestingly, the recurrence rate in our cohort was relatively low (8%), though most relapses required repeat surgical intervention and, in one case, additional radiotherapy. This underscores the need for long-term surveillance in MEN1 patients with PitNETs, even when initial management is effective. When analyzing sex-related differences, no significant associations were observed in terms of incidence, tumor size, functional status, treatment resistance, therapeutic approach, or cabergoline dose, highlighting a homogeneous behavior of MEN1-related PitNETs across sexes. These findings contrast with earlier reports suggesting a possible sex-related variability in PitNET phenotype and response to treatment but are in line with more recent evidence indicating no substantial differences between male and female patients. A large French nationwide study conducted by Le Bras and colleagues analyzed 551 MEN1 patients, of whom 202 (36.6%) had PitNETs, with a median age at diagnosis of 32 years [14]. Microadenomas were more common (57.9%) than macroadenomas (29.2%), and prolactin-secreting PitNETs represented the most frequent secreting subtype (45.5%), followed by clinically non-functioning PitNETs (36.1%). Importantly, tumor progression was rare: among 137 patients with grade I–II PitNETs, only 2.9% experienced worsening of Hardy classification over a median follow-up of 3 years, with cases mainly involving prolactin-secreting tumors or localized macroadenomas. Clinically non-functioning microadenomas, frequently detected during family screening, demonstrated particularly indolent behavior and seldom required intervention. Conversely, macroadenomas were more likely to be functional and invasive, requiring medical therapy, surgery, or radiotherapy. Overall, the study concluded that MEN1-related PitNETs are less aggressive than historically considered, with clinically non-functioning microadenomas dominating the phenotype in the modern screening era. In line with these observations, Valdés et al. recently reported a nationwide Spanish multicenter cohort of 84 MEN1 patients with long-term follow-up [15]. The mean age at diagnosis was in the mid-thirties, and most tumors were microadenomas (65.5%), with prolactin-secreting PitNETs being the most common subtype. Dopamine agonist therapy achieved biochemical control in 61% of cases, a proportion lower than typically observed in sporadic prolactinomas, suggesting reduced sensitivity to medical therapy in the MEN1 setting. Surgical resection was performed in patients with macroadenomas, and radiotherapy was rarely required. Notably, disease progression from microadenoma to invasive macroadenoma was observed in 7.2% of patients, exclusively among microprolactinomas, underscoring their long-term risk despite initial stability. As in the French cohort, no significant associations were found between tumor behavior and sex, age, or MEN1 genotype. Taken together, these studies indicate that MEN1-related PitNETs are generally characterized by a high prevalence of microadenomas, frequently detected through systematic screening, and a relatively indolent course for clinically non-functioning lesions. By contrast, functioning prolactin-secreting PitNETs remain clinically relevant because of their reduced responsiveness to dopamine agonists and potential for long-term progression, even when initially small. Our own findings are consistent with this evolving picture: we observed a balanced distribution between functioning and non-functioning tumors, a predominance of microadenomas, and a limited need for surgical intervention, mostly restricted to macroadenomas. In addition, no sex-related differences emerged in our cohort, in agreement with both multicenter series. These converging results support the notion that surveillance strategies should be individualized: long-term but less frequent monitoring is sufficient for clinically non-functioning microadenomas, while prolactin-secreting PitNETs warrant closer follow-up and may require combined therapeutic approaches, in terms of sequential medical and surgical management, given their lower medical sensitivity and risk of recurrence (Figure 1). Interestingly, some differences emerge when comparing European with extra-European cohorts. Wu and colleagues retrospectively analyzed 54 MEN1 patients with PitNETs treated at Peking Union Medical College Hospital between 2003 and 2017 [16]. The mean age at PitNET diagnosis was relatively high (53.9 years), older than in European series. Clinically non-functioning PitNETs were the most frequent subtype (48.1%), followed by prolactin-secreting PitNETs (27.8%), GH-secreting PitNETs (9.3%), ACTH-secreting PitNETs (5.6%), and mixed PitNETs (9.3%). Among prolactin-secreting tumors, bromocriptine was the primary therapy: 46.2% achieved remission, but resistance requiring surgery occurred in approximately 23% of cases. GH- and ACTH-secreting PitNETs were predominantly macroadenomas and generally treated with transsphenoidal surgery, with high remission rates (87.5% for GH, 100% for ACTH). Clinically non-functioning PitNETs—many incidentally detected through MEN1 screening—were often microadenomas (median size 4 mm), and 19 asymptomatic cases showed no progression to macroadenomas over a median 35-month follow-up. The authors emphasized that treatment prioritization in MEN1 depends on systemic involvement: thymic and enteropancreatic tumors took precedence, but functioning pituitary tumors (particularly GH- and ACTH-secreting PitNETs) required timely management, whereas clinically non-functioning lesions could be safely observed.

When compared with European cohorts and with our own data, notable differences emerge [14,15]. First, the Chinese cohort had a higher prevalence of clinically non-functioning PitNETs (48.1%) compared to the French (36%) and Spanish (34.5%) cohorts, where prolactin-secreting tumors predominated. This likely reflects systematic pituitary MRI screening and a later age at diagnosis, capturing a large number of clinically silent microadenomas. Second, dopamine agonist responsiveness in Chinese MEN1-related prolactin-secreting PitNETs was modest (≈46%), paralleling the lower efficacy observed in Valdés et al. (≈61%) and confirming that MEN1-related prolactinomas tend to be less sensitive to medical therapy than their sporadic counterparts. Third, all three studies consistently showed that clinically non-functioning microadenomas follow an indolent course, with minimal risk of progression over years of surveillance. The convergence of evidence across Europe and Asia suggests that MEN1-related PitNETs are overall less aggressive than previously assumed. However, prolactin-secreting PitNETs remain clinically challenging due to reduced pharmacological sensitivity, and functioning GH- or ACTH-secreting PitNETs require prompt active treatment. In contrast, clinically non-functioning microadenomas can be safely managed with long-term, less intensive follow-up. Moreover, a large single-institution experience from the Mayo Clinic included 268 MEN1 patients diagnosed between 1970 and 2017 [17]. PitNETs were identified in 139 individuals (51.8%), with a significantly higher prevalence in women than in men (65% vs. 35%, *p* < 0.005). The mean age at diagnosis was 36 years (range 5–80). Functioning PitNETs were more common (57%) than non-functioning ones (43%), and prolactin-secreting PitNETs represented the majority of secreting tumors (76%), followed by plurihormonal PitNETs (11%), GH-secreting (8%), and ACTH-secreting PitNETs (5%). Macroadenomas accounted for 27% of cases and were associated with local aggressiveness: they caused all visual deficits, all suprasellar extensions, and 94% of cavernous sinus invasions. Overall, 49 patients (35%) underwent transsphenoidal resection, with surgery being the first-line therapy in 84% of these cases. Remission was achieved in 81% of functional tumors after surgery, though plurihormonal PitNETs were less likely to respond. Importantly, among 52 patients with asymptomatic clinically non-functioning PitNETs followed conservatively for nearly 300 patient-years, only 5 (10%) progressed to require surgery, and none developed hypopituitarism or visual impairment before intervention. These findings strongly support conservative management for incidental, asymptomatic clinically non-functioning PitNETs in MEN1. When compared with our results and with recent European and Asian cohorts, several converging patterns emerge [14,15,16]. First, the Mayo experience confirms the predominance of prolactin-secreting tumors among functional PitNETs, but also highlights the clinical significance of plurihormonal PitNETs, which appear more aggressive and less responsive to treatment. Second, the indolent natural history of clinically non-functioning microadenomas—already emphasized in French, Spanish, and Chinese cohorts—is again confirmed by the long-term follow-up in this North American series, where surveillance proved to be a safe and effective strategy. Third, the observed sex imbalance—with a higher prevalence of pituitary disease in women—differs from our findings and from other multicenter studies, where no sex-related differences were detected; this discrepancy may reflect referral or detection biases. Taken together, the evidence from this large US series aligns with our results in underscoring that MEN1-related PitNETs are overall less aggressive than historically assumed. Surgery remains indicated mainly for symptomatic or aggressive macroadenomas, particularly those with local invasion, while clinically non-functioning microadenomas can be safely monitored. At the same time, prolactin-secreting PitNETs continue to represent the most frequent functional subtype, but with variable responsiveness to medical therapy across cohorts. Our findings of balanced functional and non-functional distribution, a predominance of microadenomas, and limited surgical indication are therefore well in line with the international experience, reinforcing the need for individualized, subtype-driven surveillance and treatment strategies in MEN1 patients.

These results contribute to the growing body of evidence describing the natural history of MEN1-related PitNETs. The homogeneous clinical course across sexes, the predominance of microadenomas and prolactin-secreting tumors, and the overall favorable treatment outcomes suggest that management strategies should primarily focus on early detection and long-term follow-up rather than on sex-specific approaches. Future multicenter prospective studies with standardized protocols are needed to confirm these observations and to further refine surveillance strategies for MEN1-associated PitNETs.

This study has several limitations. Its retrospective, single-center design may limit generalizability and introduce selection and information bias. Moreover, the long study period (2004–2025) encompasses different diagnostic, pathological, and therapeutic eras, potentially influencing the classification and management of pituitary tumors. In particular, histopathological evaluation was performed according to the standards available at the time of diagnosis, and systematic immunohistochemical assessment of pituitary transcription factors (PIT1, TPIT, and SF1), as recommended by the WHO 2022 classification of PitNETs, was not routinely available. Consequently, tumors classified as non-functioning were defined on clinical and biochemical grounds, and a definitive diagnosis of null cell PitNETs could not be consistently established, possibly leading to an overestimation of non-functioning lesions. In addition, key radiological features relevant for surgical decision-making, such as suprasellar extension, cavernous sinus invasion, and standardized visual field assessment, were not uniformly available, limiting a detailed analysis of surgical indications. Definitions of recurrence were based on evolving clinical practice and may therefore reflect heterogeneous biochemical and radiological criteria. Finally, the limited number of patients undergoing surgery, radiotherapy, or experiencing recurrence reduced the ability to perform robust multivariable analyses, and the rarity and heterogeneity of MEN1-related PitNETs constrained the statistical power of subgroup analyses. These limitations highlight the need for prospective, multicenter studies using standardized pathological and radiological criteria to better define the natural history and optimal management of PitNETs in MEN1.

## Figures and Tables

**Table 1:** Baseline Characteristics of Patients with PitNET.

Category	*n*	% (of PitNET)
Total MEN1 patients	103	
Sex	61F/42M	
Patients with PitNET	50	48.5%
Mean age at PitNET diagnosis (years)	35.1 (10–72)	
Microadenomas	30	60.0%
Macroadenomas	20	40.0%
Clinically functioning PitNET	25	50.0%
Prolactinomas (PRL)	21	42.0%
GH/PRL	2	4.0%
GH	1	2.0%
ACTH	1	2.0%
Clinically non-functioning PitNET	25	50.0%
Mean lesion size (mm)	9.4 (2–60)	
Medical therapy (DA)	20	38.0%
Cabergoline	19	36.0%
Bromocriptine	1	2.0%
Mean weekly DA dose (mg)	1.32 (0.5–4.5)	
Endoscopic neurosurgery	7	14.0%
Recurrence	4	8.0%
Medical therapy after recurrence	1	
Repeat neurosurgery	3	
Stereotactic radiotherapy	1	

## Data Availability

The data presented in this study are available on request from the corresponding author.

## Abbreviations

The following abbreviations are used in this manuscript:

MEN1	Multiple endocrine neoplasia type 1
PitNET	Pituitary neuroendocrine tumor
GTE	French Group of Endocrine Tumors
BMI	Body mass index
MRI	Magnetic resonance imaging
CT	Thoracic computed tomography
